# Regional brain serotonergic activity as an indicator of chronic stress and compromised welfare in fish

**DOI:** 10.3389/fendo.2026.1736618

**Published:** 2026-02-11

**Authors:** Christina Sørensen, Judit Vas, Ole Folkedal, Samantha Bui, Mette Remen, Mikkel Gunnes, Sara Calabrese, Louise O. M. Wedaa, Evelina A. L. Green, Øyvind Øverli, Erik Höglund

**Affiliations:** 1 Norwegian Institute for Water Research, Bergen, Norway; 2 Department of Animal and Aquacultural Sciences, Faculty of Biosciences, Norwegian University of Life Sciences, Ås, Norway; 3 Animal Welfare Research Group, Institute of Marine Research, Matredal, Norway; 4 Aquaculture Department, SINTEF Ocean AS, Trondheim, Norway; 5 Center for Coastal Research, University of Agder, Kristiansand, Norway

**Keywords:** aquaculture, Atlantic salmon, brain stem, cortisol, serotonin, stress, telencephalon, welfare

## Abstract

**Background:**

Chronic stress in aquaculture poses major challenges to fish welfare, health, and productivity. Detecting compromised welfare early requires validated biomarkers that link operational welfare indicators (OWIs) to underlying neuroendocrine states, including allostatic load and stress coping capacity. The central serotonergic system is a promising candidate for such validation, given its conserved role in stress regulation across vertebrates.

**Methods:**

We sampled 40 Atlantic salmon (*Salmo salar*) from research-scale sea cages spanning a wide range of welfare states under production-relevant conditions. Fish were exposed to acute crowding during routine rearing operations immediately prior to sampling. Plasma cortisol was measured, along with serotonin (5-HT) concentration and turnover, proxied by 5-hydroxyindoleacetic acid (5-HIAA) and the 5-HIAA/5-HT ratio, in the brain stem and telencephalon. Associations among 5-HTergic variables, welfare indicators (OWI sum, scale loss, condition factor), and acute stress metrics (crowding time, cortisol) were analysed using principal component analysis (PCA), multivariate analysis of variance (MANOVA), and *post hoc* linear models.

**Results:**

Brain stem 5-HT concentration and turnover were consistently associated with welfare-related indicators and loaded strongly with these variables in multivariate space, indicating sensitivity to cumulative stress and compromised welfare. In contrast, telencephalic 5-HT turnover aligned primarily with acute stress metrics rather than welfare state. MANOVA and *post hoc* analyses confirmed these region-specific associations. A significant interaction for telencephalic 5-HIAA revealed that fish in poorer welfare states exhibited a blunted 5-HTergic response to acute stress compared with individuals in better welfare condition, consistent with reduced stress responsiveness under chronic strain.

**Conclusion:**

Our findings demonstrate a clear regional dissociation in central 5-HTergic stress processing in Atlantic salmon: brain stem 5-HT activity reflects chronic stress burden and welfare state, whereas telencephalic 5-HT turnover reflects acute stress reactivity conditional on baseline welfare. These results support the use of region-specific 5-HTergic measures as biologically grounded markers distinguishing chronic welfare compromise from functional acute stress responses in aquaculture, while emphasizing caution when interpreting acute stress reactivity in chronically stressed individuals.

## 1 Introduction

Global demand for sustainable animal protein has driven rapid growth and innovation in aquaculture, making it one of the fastest-expanding food production sectors worldwide (1–3). Salmon farming has evolved into a highly efficient and technology-driven industry, characterized by large-scale operations and the use of advanced control systems for production, monitoring, and disease management. Despite this progress, the intensification of aquaculture has brought with it significant challenges related to mortality, fish health and welfare (4). High-density rearing, handling procedures, malnutrition, parasite outbreaks, and other stressors can compromise welfare and lead to reduced growth, impaired immune function, and increased mortality (5–9).

Ensuring high welfare standards for farmed fish is not only an ethical imperative but also critical for maintaining biological performance and consumer trust. However, detecting compromised welfare early enough to allow for preventative intervention remains a major challenge. Most traditional welfare assessments rely on operational welfare indicators (OWIs) that become apparent only after long-term stress, major injury, or salient impairment has occurred, such as visible wounds, aberrant behaviour, or low condition factor. These indicators, while useful, are often retrospective and may be insensitive to the early stages of chronic stress (10, 11). Furthermore, most monitoring systems assess group-level welfare and fail to capture significant individual variation in stress coping, which has important implications for both welfare and productivity (12–14). To address this limitation, increasing attention is being directed toward the development and validation of biologically relevant welfare indicators that can detect the onset of stress and compromised welfare in a timely and potentially non-invasive manner (15–17). It is of critical importance to validate such welfare indicators so that they have a solid foundation in stress physiology and neurobiology (18). Well validated welfare indicators that are accurate, practical and minimally invasive will be important to support the refinement of sensor technologies and AI-based monitoring tools that aim to assess welfare at the individual level in real time (19–21).

A promising approach to validating welfare indicators is to anchor their outputs to neuroendocrine markers of allostatic state and stress coping ability. Allostatic state refers to the cumulative physiological burden imposed by repeated or prolonged stressors, reflecting how organisms maintain stability through adaptive change (22, 23). The central 5-HTergic system is a strong candidate for such validation. 5-HT (serotonin) is an evolutionarily conserved neuromodulator that regulates stress responsiveness, aggression, and social behaviour across vertebrates, including teleost fish (24–26). In fish, central 5-HT activity is commonly assessed by measuring 5-HT and its primary metabolite 5-hydroxyindoleacetic acid (5-HIAA). Following synaptic release, 5-HT is rapidly taken up from the extracellular space and metabolized to 5-HIAA; accordingly, 5-HIAA concentration and the 5-HIAA/5-HT ratio are widely used proxies for 5-HT turnover (27).

Turnover of 5-HT increases reliably in response to acute stress and undergoes longer-term modulation following repeated or chronic stress exposure (27–30). These properties capture both transient neurochemical responses to short-term challenges and persistent alterations linked to coping capacity, offering a mechanistic basis for distinguishing acute from chronic stress states. However, most studies of brain 5-HTergic activity in fish have been conducted under controlled laboratory conditions or have focused on mechanistic stress physiology rather than on direct associations with operational welfare indicators (OWIs) in production-relevant aquaculture settings. In the present study, we have examined brain 5-HT activity in Atlantic salmon (*Salmo salar*) under production-relevant rearing conditions, relating these measurements to welfare state based on commonly used OWIs and to acute stress caused by crowding. We have assessed the 5-HTergic activity in specific brain regions to evaluate its sensitivity to both acute and cumulative stress exposure. Our aim has been to further establish 5-HT turnover as a biomarker of stress and compromised welfare, and to determine whether it can be meaningfully linked to established welfare indicators currently used in the aquaculture industry. We also sought to identify distinct regional brain 5-HT activation patterns that differentiate chronic from acute stress, investigating these 5-HTergic activities as integrative and biologically relevant markers in Atlantic salmon.

## 2 Materials and methods

### 2.1 Subjects and housing

Atlantic salmon *(Salmo salar*) were produced at the Institute of Marine Research’s facilities in Matre, Norway, according to standard production practices. In April 2023, approximately 12000 fish were transported to the Institute of Marine Research’s Austevoll Research Station, where they were released into four sea cages (12 x 12 m, 14 m deep), providing a semi-commercial setting for the experiment. The facility is a registered site for animal experiments, and the use of animals in this experiment complied with all regulations and guidelines set by the Norwegian Food Safety Authority. As all procedures and treatment of the fish were conducted within normal aquaculture operational practices, the experiments were determined not to require special ethical approval.

The fish were sampled on 10 October 2023 as part of ongoing rearing operations, specifically during routine sampling for ectoparasitic sea lice registration, as are mandatory for all Norwegian aquaculture facilities. A total of 40 fish were collected from four research-scale sea cages. The mean fish weight across cages was 2068 g, and the ambient water temperature during sampling was 12.8 °C at 0.5 m depth.

### 2.2 Sampling

Fish were captured using a rectangular seine net (12 × 7 m), which was pulled to crowd a group of fish near the surface. The fish remained confined within the net for up to 1.5 h before sampling. Focal fish were identified and individually captured using a hand net, then humanely euthanized with MS-222 (0.5 g/L). Fish were selected to represent the full range of welfare states observable from the cage side (i.e., based on visual assessment of size, condition, and external damage).

The number of salmon louse (*Lepeophtheirus salmonis*), an ectoparasite known to influence chronic stress at high infestation levels, was recorded for each fish according to standard manual louse counting procedures (31). Fork length and body weight were measured, and each fish was photographed on both sides. Welfare was assessed according to the Laksevel protocol (32), which includes scoring of *first impression*, sp*inal deformities, emaciation, sexual maturation, scale loss, skin bleeding, body wounds, snout wounds, jaw deformities, eye clouding, eye damage*, and *operculum condition*. Each variable was rated on a 0–3 scale, where 0 indicates no apparent damage and 3 indicates severe damage. *Skin bleeding, snout damage*, and *gill status* were scored on-site, while the remaining variables were evaluated from photographs after sampling (32). For statistical analysis, sums of scores were calculated and designated as the operational welfare indicator (OWI) sum. Photographs were also analyzed for percentage scale loss following the method described by (31). Condition factor (K) was calculated as:


$$K=\;\frac{W}{L^{3}}*100$$

where W is weight in g and L is length in cm.

Approximately 1 mL of blood was sampled from the caudal vein using a heparinized syringe. For each individual fish, the time from onset of crowding (i.e., initiation of net pulling) to blood sampling was recorded. Blood samples were immediately transferred to tubes and centrifuged at 1500 g for 10 min at 4 °C. Blood plasma samples were transferred to clean tubes, frozen on dry ice and stored at -80 °C. Brains were dissected out, and telencephalon and brain stem were separated. Individual brain regions were wrapped in aluminium foil, frozen on dry ice and stored at -80 °C. Finally, sex was determined after opening the abdomen.

### 2.3 Analysis of plasma cortisol and 5-HT neurochemistry

Cortisol in blood plasma was analyzed using a commercially available DetectX^®^ cortisol enzyme immunoassay kit (Arbor Assays, Ann Arbor, MI, USA) following the manufacturer’s protocol. The absorbance of the prepared ELISA plate was read in a Multiskan™ FC (Thermo Scientific™) plate reader at 450 nm and cortisol concentrations were calculated using four-parameter logistic regression.

Frozen brain samples were homogenized in 4% (w/v) ice-cold perchloric acid containing 0.2% EDTA and 94.2 ng/mL of 3,4-dihydroxybenzylamine hydrobromide (DHBA) as internal standard, by using an MSE 100W ultrasonic disintegrator. The samples were thawed on ice, and centrifuged at 17000 rpm for 5 min. From the supernatant, 5-HT and 5-HIAA were quantified using High-performance liquid chromatography (HPLC) with electrochemical detection. The HPLC system consisted of a solvent-delivery system (Shimadzu, LC-10AD), an auto injector (Famos, Spark), a reverse-phase column (4.6 mm × 100 mm, Hichrom, C18, 3.5 μm) and an ESA Coulochem II detector (ESA, Bedford, MA, USA) with two electrodes, at −40 and +320 mV. A conditioning electrode (ESA 5020), with a potential of +400 mV, was employed before the analytical electrodes, to oxidize any possible contaminants present. The mobile phase consisted of 86.25 mM/L of sodium phosphate, 1.4 mM/L of sodium octyl sulfate and 12.26 μM/L of EDTA in deionized (resistance 18.2 MΩ) water containing 7% acetonitrile brought to a pH of 3.1 with phosphoric acid. Samples were quantified by comparison with standard solutions of known concentrations and corrected for recovery of the internal standard using the HPLC software (CSW, DataApex Ltd, Czech Republic).

For a small number of individuals, tissue loss during sampling resulted in missing cortisol or 5-HT measurements; these cases were excluded from the relevant analyses.

### 2.4 Data analysis

The data were handled and analysed in R (4.4.1) (33) using RStudio (2025.09.1 Build 401 (34)). Relationships between variables were visualized using scatter plots with least-squares linear regression lines and 95% confidence intervals (**Supplementary Figures S1–S4**). For transparency, coefficients of determination (R²) and associated p-values for individual welfare, acute stress, and 5-HTergic variables are reported in the figures; these correlations are presented descriptively and are not adjusted for multiple testing.

Due to deviation from normal distribution, scale loss, 5-HIAA concentrations, and 5-HIAA/5-HT ratios were log-transformed when appropriate, whereas cortisol concentration was square-root transformed. One telencephalon 5-HIAA value (and the corresponding 5-HIAA/5-HT value) was removed, as it exceeded four standard deviations from the mean.

To further investigate relationships among variables and reduce data dimensionality, two principal component analyses (PCA) were performed (35) using the FactoMineR package (36). PCA1 was used as an exploratory dimensionality-reduction approach to examine the overall structure of the dataset and identify patterns of covariance among welfare indicators, acute stress metrics, and central 5-HTergic variables. Variables included in PCA1 were weight, length, condition factor, sex, OWI sum, scale loss, lice count, and 5-HTergic measures from the brain stem and telencephalon (5-HT, 5-HIAA, and 5-HIAA/5-HT). PCA2 was conducted to derive composite independent variables for use in subsequent regression-based analyses and to minimize multicollinearity. Variables included in PCA2 were condition factor, scale loss, OWI sum, crowding time, and cortisol. All PCA results were visualized using the factoextra package (37). Before running the models, data were scaled and centered, and missing values were imputed using the missMDA package (38).

To examine whether welfare state and acute stress exposure were statistically associated with multivariate patterns of 5-HTergic activity, a multivariate analysis of variance (MANOVA) was fitted with dimensions from PCA2 representing welfare and acute stress as independent variables, and all monoamine measures (brain stem 5-HT, 5-HIAA (log-transformed), and 5-HIAA/5-HT ratios (log-transformed), and telencephalon 5-HT, 5-HIAA, and 5-HIAA/5-HT ratios) as dependent variables. MANOVA was used as the primary inferential analysis to test for overall multivariate effects across correlated neurochemical measures.

*Post hoc* univariate linear models were fitted for each monoamine variable, following significant multivariate effects, and Holm’s method was applied to adjust p-values for multiple testing across outcomes within each predictor (welfare, acute stress and welfare x acute stress). Model estimates are reported as effect sizes with 95% confidence intervals, t-statistics, raw p-values, and Holm-corrected p-values in **Supplementary Table S1**. Model assumptions were evaluated for all multivariate and univariate analyses. Residuals were inspected visually for normality and homoscedasticity, and influential observations were assessed. No substantial violations affecting model interpretation were detected.

**Figure 1:**
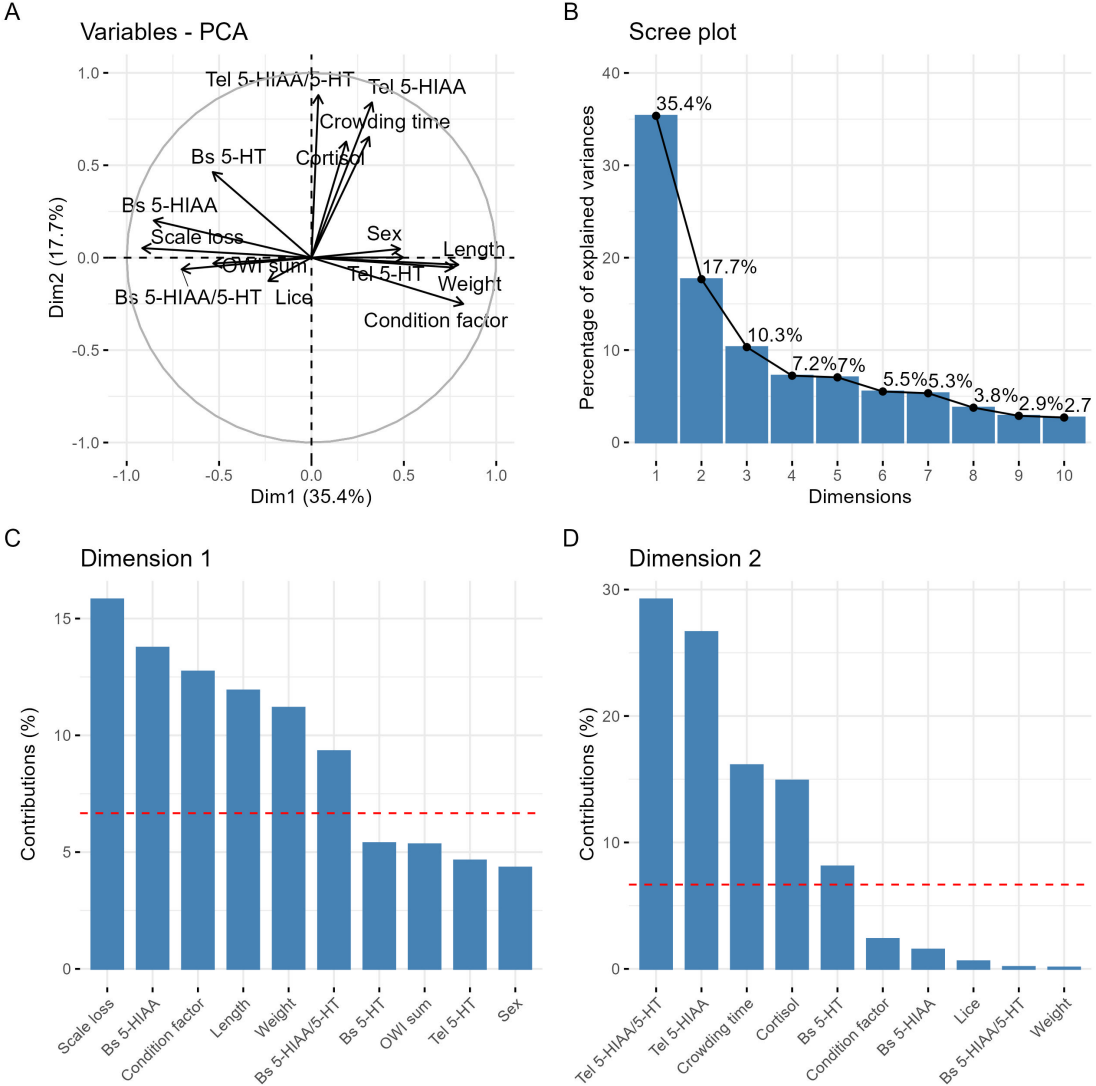
Results from the exploratory principal component analysis (PCA1) including welfare indicators, acute stress measures, and 5-HTergic variables. **(A)** Variable plot of PCA1 dimensions 1 and 2, where arrows represent variable loadings. **(B)** Scree plot showing the proportion of variance explained by the first ten PCA1 dimensions. **(C, D)** Contributions of individual variables to PCA1 dimension 1 **(C)** and dimension 2 **(D)**. Red dashed lines indicate the expected contribution if all variables contributed equally.

**Figure 2:**
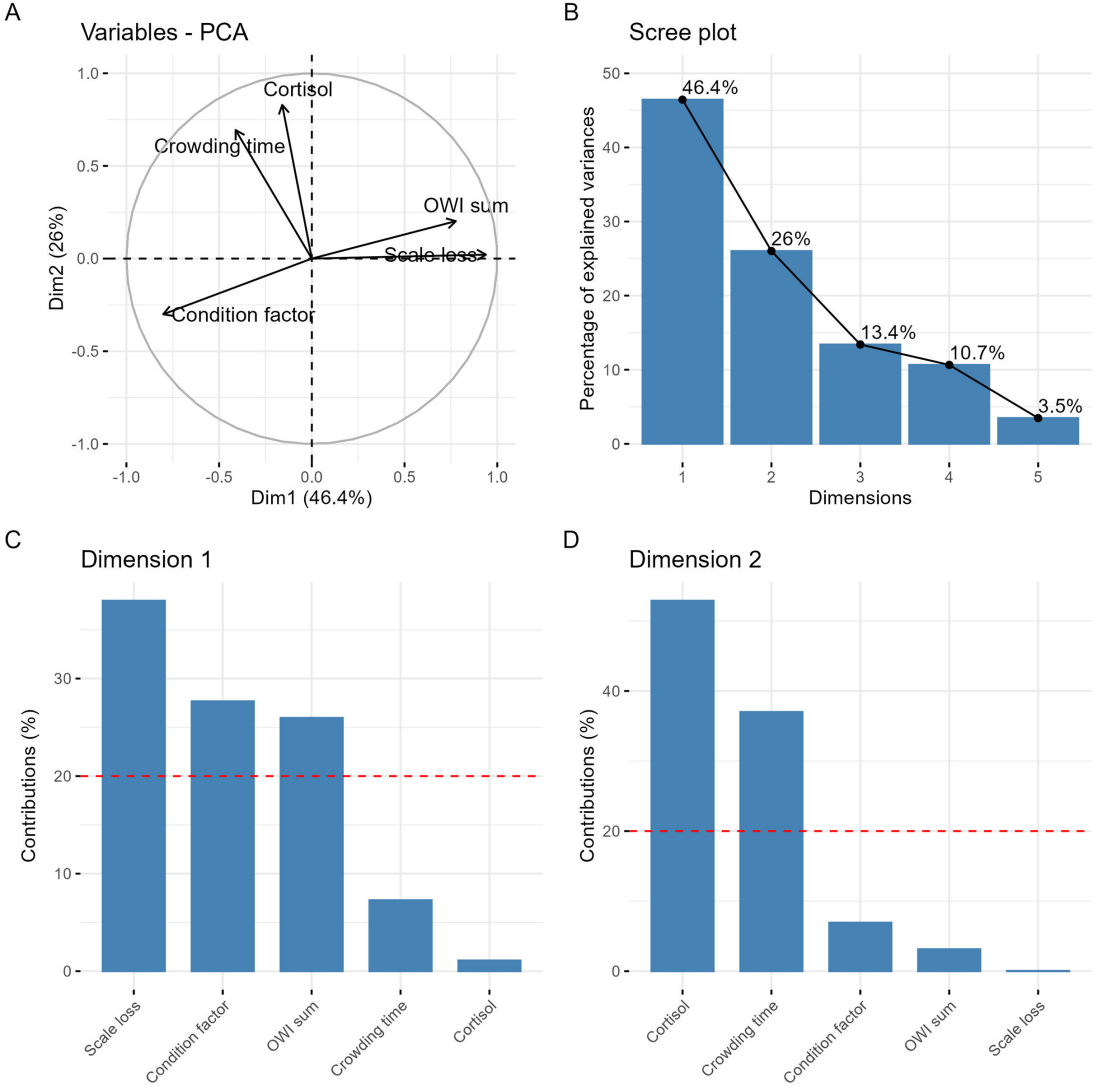
Results from the second principal component analysis (PCA2) used to derive composite dimensions of welfare and acute stress. **(A)** Variable plot of PCA2 dimensions 1 and 2, where arrows represent variable loadings. **(B)** Scree plot showing the proportion of variance explained by the first ten PCA2 dimensions. **(C, D)** Contributions of individual variables to PCA2 dimension 1 **(C)** and dimension 2 **(D)**. Red dashed lines indicate the expected contribution if all variables contributed equally.

**Figure 3:**
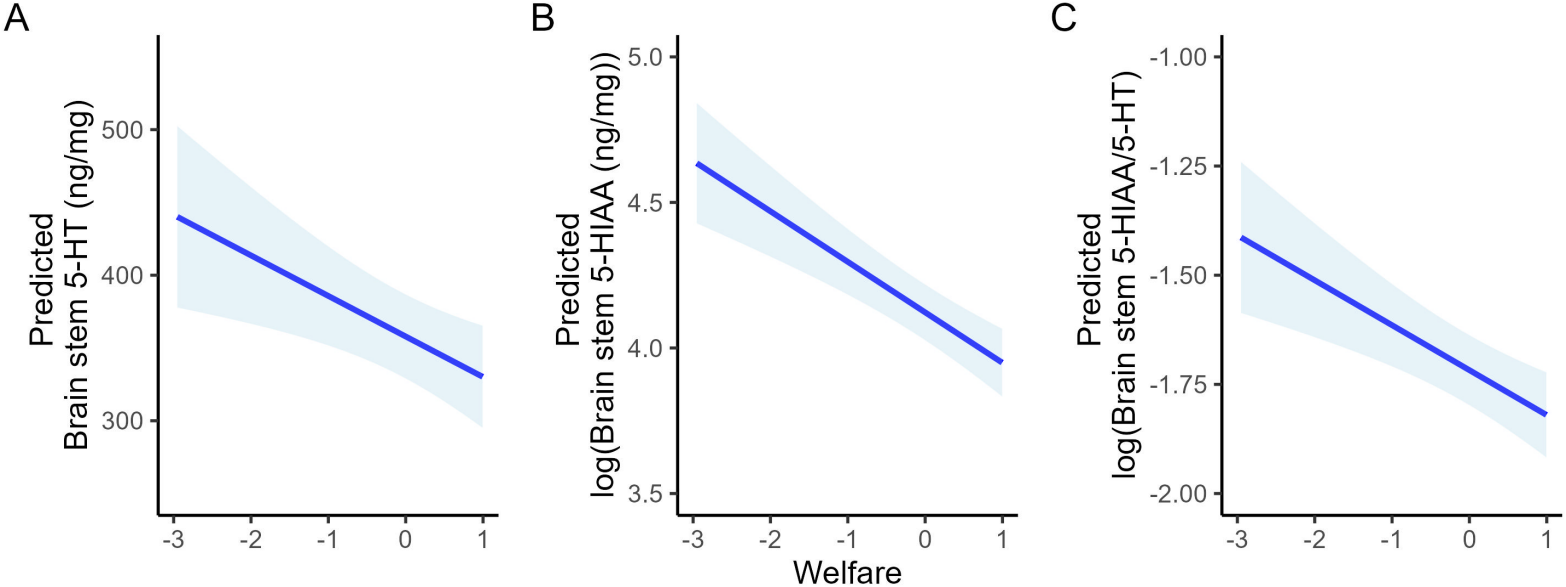
Model-based predicted values of brain stem 5-HTergic activity across representative values of welfare (PCA2 dimension 1), with acute stress held constant at its mean. Panels show predicted **(A)** brain stem 5-HT concentration, **(B)** log of brain stem 5-HIAA concentration, and **(C)** log of brain stem 5-HIAA/5-HT ratio. Lines represent fitted values from the *post hoc* linear models, and shaded areas denote 95% confidence intervals. Predictions are intended to illustrate overall response patterns and associated uncertainty rather than precise quantitative effect sizes.

To aid interpretation of statistically supported effects, model-based predicted values and 95% confidence intervals were calculated from the fitted linear models. Predictions were generated only for 5-HTergic variables showing significant associations with welfare or acute stress. Specifically, predicted values were calculated for brain stem 5-HT, 5-HIAA, and 5-HIAA/5-HT across representative values of welfare while holding acute stress constant at its mean (**Figure 1**), and for telencephalon 5-HIAA and 5-HIAA/5-HT across representative values of acute stress while holding welfare constant at its mean (**Figure 2**). These predictions are presented to illustrate overall patterns of association and uncertainty rather than to imply precise quantitative effect sizes. The same approach was applied to the significant interaction involving telencephalon 5-HIAA, examining its response across a sequence of acute stress values at representative levels of welfare (**Figure 3**).

## 3 Results

Atlantic salmon (n = 40) spanning a wide range of welfare states were sampled from four semi-commercial sea cages, with individuals selected to capture variation in body condition and external damage (**Table 1**).

**Table 1:** Summary of population sample characteristics showing the range and mean ± standard deviation for all measured variables.

Variable	Range	Mean ± SD	Unit/Notes
Weight	210 – 3425	1490 ± 902	g
Length	310 – 620	473 ± 89	mm
Condition factor	0.61 – 1.71	1.20 ± 0.24	Fulton’s K = 100 × (W/L³)
OWI sum	3 – 14	6.6 ± 2.8	Composite welfare score
Scale loss	1.1 – 49.9	8.4 ± 10.5	% of body surface affected
Sea lice	0 – 18	4.6 ± 4.2	Individuals per fish
Crowding time	17 – 84	52.4 ± 16.9	min
Plasma cortisol	0.6 – 293.9	98.4 ± 74.1	ng/mL
Brain stem 5-HT	164 – 687	365 ± 105	ng/mg tissue
Brain stem 5-HIAA	30 – 217	69 ± 36	ng/mg tissue
Brain stem 5-HIAA/5-HT	0.11 – 0.44	0.19 ± 0.06	Ratio (5-HT turnover)
Telencephalon 5-HT	2025 – 4867	3664 ± 661	ng/mg tissue
Telencephalon 5-HIAA	310 – 1738	713 ± 258	ng/mg tissue
Telencephalon 5-HIAA/5-HT	0.06 – 0.20	0.11 ± 0.04	Ratio (5-HT turnover)

**Figure 4:**
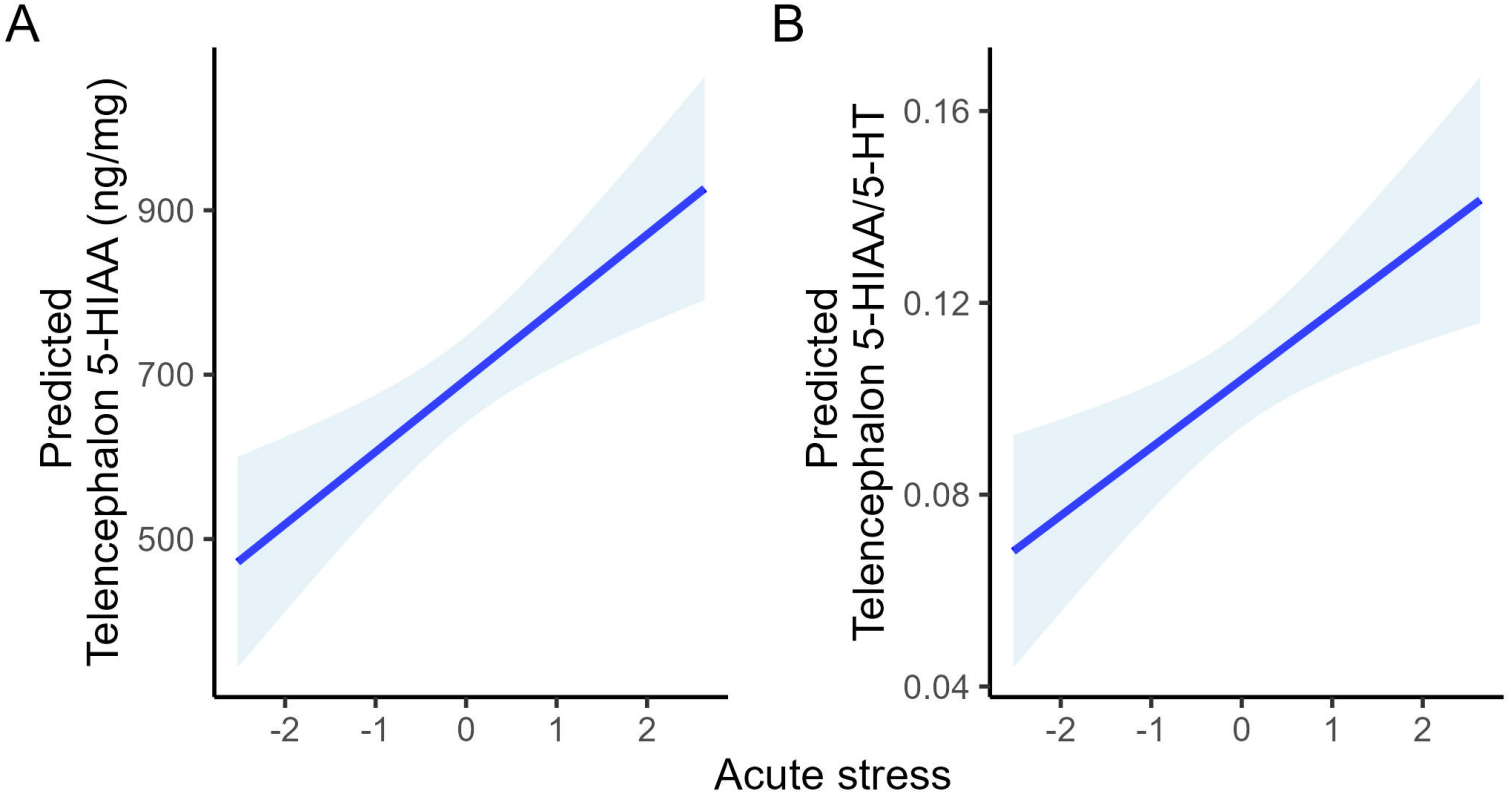
Model-based predicted values of telencephalic 5-HTergic activity across representative values of acute stress (PCA2 dimension 2), with welfare held constant at its mean. Panels show predicted **(A)** telencephalic 5-HIAA concentration and **(B)** telencephalic 5-HIAA/5-HT ratio. Lines represent fitted values from the *post hoc* linear models, and shaded areas denote 95% confidence intervals. Predictions are intended to illustrate overall response patterns and associated uncertainty rather than precise quantitative effect sizes.

Exploratory principal component analysis (PCA1) revealed a clear separation between welfare-related and acute stress-related variables (**Figure 4**). The first two dimensions together explained 53.1% of the total variance and reflected biologically distinct stress domains. Dimension 1 was primarily associated with welfare indicators, including scale loss, condition factor, and OWI sum, and aligned closely with brain stem 5-HTergic activity (5-HIAA and 5-HIAA/5-HT). In contrast, dimension 2 was dominated by acute stress metrics (crowding time and plasma cortisol) and corresponded most strongly with telencephalic 5-HTergic turnover (5-HIAA and 5-HIAA/5-HT). These patterns indicate a regional dissociation in 5-HTergic responses, with brain stem activity reflecting longer-term welfare status and telencephalic activity reflecting acute stress exposure.

**Figure 5:**
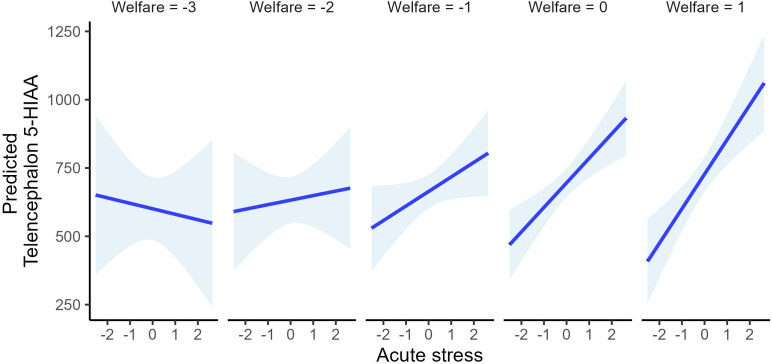
Model-based predicted telencephalic 5-HIAA concentrations across a sequence of acute stress values (PCA2 dimension 2) at representative levels of welfare (PCA2 dimension 1). Separate panels depict predictions for welfare scores of -3, -2, -1, 0, and 1. Lines represent fitted values from the *post hoc* linear model, and shaded areas denote 95% confidence intervals. The figure illustrates a welfare-dependent modulation of the telencephalic 5-HTergic response to acute stress, with progressively attenuated responses at lower welfare levels. Predictions are intended to illustrate overall response patterns and associated uncertainty rather than precise quantitative effect sizes.

To formalize these biological dimensions for subsequent analyses, a second PCA (PCA2) was conducted using welfare and acute stress indicators only. This analysis yielded two interpretable composite axes explaining 72.4% of the variance (**Figure 5**). Dimension 1 captured welfare state, with positive loadings for scale loss and OWI sum and a negative loading for condition factor; for clarity, this axis was inverted for use in further analysis so that higher values represent better welfare. Dimension 2 captured acute stress, driven by crowding time and cortisol, reflecting both stressor intensity and physiological response. These two PCA2 dimensions were denoted “welfare” and “acute stress” and were used as independent variables in all subsequent multivariate and univariate analyses.

Multivariate analysis of variance demonstrated that both welfare and acute stress were significantly associated with overall patterns of brain 5-HTergic activity, with a further significant interaction between the two, as reflected by Pillai’s trace (welfare: V = 0.517, F_(6, 30)_ = 5.36, p < 0.001; acute stress: V = 0.517, F_(6, 30)_ = 5.35, p < 0.001; welfare × acute stress: V = 0.360, F_(6, 30)_ = 2.81, p = 0.027). *Post hoc* linear models identified the sources of these multivariate effects. Brain stem 5-HT concentration and turnover (5-HIAA and 5-HIAA/5-HT) were significantly associated with welfare state, whereas telencephalic 5-HT turnover (5-HIAA and 5-HIAA/5-HT) were primarily associated with acute stress (**Table 2**; **Supplementary Table S1**).

**Table 2:** Summary of *post hoc* univariate linear model results for 5-HTergic variables (brain stem and telencephalon 5-HT, 5-HIAA, and 5-HIAA/5-HT) with model terms representing Welfare (PCA2 dimension 1), Acute stress (PCA2 dimension 2), and their interaction (Welfare × Acute stress), fitted following MANOVA.

Dependent variable	Terms	Holm p-value	Significance
Brain stem 5-HT			
	Welfare	0.02	*
	Acute stress	0.1125	
	Welfare × acute stress	0.6095	
log(Brain stem 5-HIAA)			
	Welfare	0	***
	Acute stress	0.1125	
	Welfare × acute stress	0.3778	
log(Brain stem 5-HIAA/5-HT)			
	Welfare	0.0013	**
	Acute stress	1	
	Welfare × acute stress	0.6095	
Telencephalon 5-HT			
	Welfare	0.0898	(*)
	Acute stress	1	
	Welfare × acute stress	0.5975	
Telencephalon 5-HIAA			
	Welfare	0.0511	(*)
	Acute stress	0.0005	***
	Welfare × acute stress	0.0061	**
Telencephalon 5-HIAA/5-HT			
	Welfare	0.4348	
	Acute stress	0.0032	**
	Welfare × acute stress	0.2335	

Reported p-values are Holm-corrected for multiple testing within each term separately. Full model statistics, including effect sizes, 95% confidence intervals, *t*(df) values, raw and Holm-corrected p-values, are provided in **Supplementary Table S1**. Significance levels: ***p < 0.001, **p < 0.01, *p < 0.05, (*) p < 0.1.

These relationships are illustrated by model-based predictions showing monotonic changes in brain stem 5-HTergic activity across representative welfare levels (**Figure 1**) and in telencephalic 5-HTergic turnover across increasing acute stress exposure (**Figure 2**), highlighting that worsening welfare is associated with elevated brain stem 5-HTergic activity, whereas increasing acute stress selectively engages telencephalic 5-HTergic turnover.

Importantly, telencephalic 5-HIAA showed a significant interaction between welfare and acute stress. Model predictions revealed that individuals in good welfare condition mounted a robust, dose-dependent increase in telencephalic 5-HIAA in response to increasing acute stress, whereas this response was progressively attenuated in individuals with poorer welfare and absent in those with the lowest welfare scores (**Figure 3**). This pattern indicates a blunted central 5-HTergic response to acute challenge in chronically compromised individuals.

## 4 Discussion

### 4.1 Main findings: regional 5-HTergic markers distinguish chronic and acute stress

In this study, we investigated whether central 5-HTergic activity in discrete brain regions can distinguish chronic, welfare-related stress from acute stress exposure in farmed Atlantic salmon under production-relevant conditions. Our results demonstrate a clear regional dissociation in 5-HTergic responses: brain stem 5-HT concentration and turnover were strongly associated with long-term welfare state, whereas telencephalic 5-HT turnover primarily reflected acute stress exposure. Importantly, we also identified a significant interaction between welfare and acute stress, indicating that individuals in poorer welfare states exhibit a blunted telencephalic 5-HTergic response to acute challenges. These findings extend controlled experimental work by demonstrating that this regional dissociation in 5-HTergic stress processing is preserved under the complex and variable conditions of production-relevant aquaculture.

Interpreting these findings in light of the composite welfare and acute stress dimensions highlights biologically meaningful patterns. Individuals with higher welfare scores were characterized by better body condition, fewer external injuries, and lower OWI scores, whereas higher acute stress scores corresponded to longer exposure to crowding and elevated plasma cortisol. Brain stem 5-HTergic activity tracked the cumulative physiological burden associated with reduced welfare, whereas telencephalic 5-HTergic turnover responded dynamically to acute stressors. This functional separation supports the interpretation that distinct components of the central 5-HTergic system encode different temporal aspects of the stress experience.

This regional dissociation is consistent with the anatomical organization of the teleost 5-HTergic system (25). In fish, 5-HTergic cell bodies are located primarily in the inferior and superior raphe nuclei of the brain stem, which serve as the main source of central 5-HT production. These nuclei give rise to ascending projections that innervate forebrain regions, including the telencephalon, which lacks intrinsic 5-HT synthesis. Brain stem raphe neurons are rich in autoreceptors that regulate firing and release, supporting their role in integrating sustained stress input over time, whereas telencephalic 5-HTergic activity reflects downstream modulation of acute stress processing. This anatomical organization provides a mechanistic basis for the observed sensitivity of brain stem 5-HTergic activity to chronic stress load and the more labile, stress-responsive nature of telencephalic 5-HT turnover.

The regional differentiation observed here is consistent with previous experimental work on stress neurobiology in fish. Øverli et al. (29) provided early evidence that telencephalic 5-HTergic activity responds rapidly to acute social stress and normalizes once the challenge resolves, whereas brain stem 5-HTergic activation emerges only after prolonged stress exposure. In their social hierarchy experiments, subordinate fish showed sustained elevations in telencephalic 5-HT turnover and delayed increases in brain stem 5-HT turnover, whereas dominant fish exhibited only transient telencephalic activation after hierarchy formation. This temporal dissociation closely mirrors the patterns observed in the present study.

More recent studies under aquaculture-relevant conditions further support this interpretation. Vindas et al. (30) demonstrated that growth-stunted Atlantic salmon with low welfare status exhibited elevated basal brain stem 5-HT, 5-HIAA, and 5-HIAA/5-HT ratios compared with individuals in better welfare condition. When exposed to an additional acute stressor, these chronically stressed fish failed to mount further increases in brain stem 5-HTergic activity, whereas fish in normal welfare condition showed clear stress-induced responses. Although telencephalic 5-HT activity was not measured in that study, our observation of a blunted telencephalic 5-HIAA response in low-welfare individuals extends these findings and provides additional insight into region-specific exhaustion of stress responsiveness.

This interpretation is further supported by later work showing that chronic stress regimes in Atlantic salmon can produce interaction effects between chronic and acute stress in both brain stem and telencephalic 5-HTergic activity (39). Subtle differences in 5-HTergic responses between studies likely reflect variation in experimental design, including life stage, temperature, stressor type, and duration, but collectively point to a common principle: chronic stress alters the capacity of the 5-HTergic system to respond adaptively to new challenges. Taken together, these findings support the concept of allostatic overload in fish. Chronic stress appears to push individuals toward physiological limits, resulting in attenuated neurochemical and endocrine responses to additional stressors. Such blunting of stress responsiveness has been observed in multiple fish species and experimental contexts (30, 39–41) and is increasingly recognized as a key feature of compromised welfare.

### 4.2 Implications for welfare assessment in aquaculture

The present findings have important implications for welfare assessment in intensive aquaculture. The robust association between welfare state and brain stem 5-HT concentration and turnover identifies a neurobiologically grounded marker of chronic stress that is relatively insensitive to concurrent acute stress exposure. This stability suggests that brain stem 5-HTergic activity may provide an integrative measure of cumulative welfare burden, capturing sublethal physiological strain before overt injuries or pronounced reductions in condition factor become apparent, and remaining informative even when fish are exposed to acute stress at the time of sampling.

Notably, elevated brain stem 5-HTergic activity has been detected within 24 hours of chronic stress exposure in experimental settings (29), indicating that this marker may respond earlier than many traditional operational welfare indicators. Together, these observations suggest that brain stem 5-HTergic activity reflects early physiological responses to sustained stress and may therefore serve as a reference measure against which operational welfare indicators could be evaluated or validated, rather than implying direct application in routine monitoring.

At the same time, our results highlight an important caveat in the interpretation of acute stress responses. Telencephalic 5-HTergic turnover clearly reflects acute stress exposure in individuals with good welfare, but this response becomes progressively attenuated as welfare declines. In chronically stressed fish, reduced telencephalic 5-HTergic responses are likely to reflect physiological exhaustion rather than resilience or successful coping. Consequently, reliance on acute stress markers alone may underestimate total stress burden in populations with compromised welfare.

Together, these findings support a framework in which brain stem and telencephalic 5-HTergic activity provide complementary information: brain stem measures reflect chronic stress load and welfare state, whereas telencephalic measures reflect acute stress responsiveness conditional on baseline welfare. This distinction is critical for the development of biologically meaningful welfare indicators in aquaculture.

### 4.3 Limitations and future directions

The present study is observational and based on spontaneously occurring variation in welfare state under authentic aquaculture conditions. As such, we cannot determine whether the observed 5-HTergic patterns comprise a consequence of compromised welfare, a predisposing factor, or a combination of both. Pre-existing individual differences in stress coping styles and 5-HTergic regulation are well documented in fish and may influence both welfare trajectories and neurochemical responses (12, 13, 42, 43).

In addition, the use of composite dimensions necessarily reduces resolution at the level of individual welfare indicators or stressors with respect to their specific impact on 5-HT neurobiology. While this approach improves interpretability and minimizes multicollinearity, controlled experiments will be required to quantify the contribution of individual stressors and to assess the generalizability of the observed relationships across seasons, temperatures, life stages, and production contexts. Finally, although our focus was on the brain stem and telencephalon, other brain regions, particularly the hypothalamus, play a central role in regulation of the hypothalamus-pituitary-interrenal (HPI) axis and should be considered in future studies.

### 4.4 Conclusion

In summary, we demonstrate that brain stem 5-HT concentration and turnover align with chronic stress burden and compromised welfare in Atlantic salmon, whereas telencephalic 5-HT turnover reflects acute stress reactivity in a welfare-dependent manner. Chronic stress appears to blunt telencephalic 5-HTergic responsiveness to acute stress, consistent with an allostatic overload model. By linking operational welfare indicators to region-specific neurobiological mechanisms, this work provides a biological framework for interpreting welfare indicators in relation to central stress physiology and contributes to the validation of welfare assessment approaches used in aquaculture.

## Data Availability

The raw data supporting the conclusions of this article will be made available by the authors, without undue reservation.
