# Supplementary material for: Regional brain serotonergic activity as an indicator of chronic stress and compromised welfare in fish
Source: Front Endocrinol (Lausanne). 2026 Feb 11;17:1736618. doi: 10.3389/fendo.2026.1736618 (PMC12932212; doi:10.3389/fendo.2026.1736618)
Supplement: Supplementary file 1 [file DataSheet1.pdf]

Supplementary Material

Dependent variable	Terms	Estimate	95% confidence interval	t statistic (df)	Raw p-value	Holm p-value	Significance
Brain stem 5-HT							
	Welfare	-28.4211	[-47.6911, -9.1511]	-2.99 (36)	0.005	0.02	*
	Acute stress	29.0828	[3.3006, 54.8651]	2.29 (36)	0.0281	0.1125	
	Welfare × acute stress	7.0208	[-10.1146, 24.1563]	0.83 (36)	0.4115	0.6095	
log(Brain stem 5-HIAA)							
	Welfare	-0.1718	[-0.2339, -0.1097]	-5.61 (36)	0	0	***
	Acute stress	0.0907	[0.0076, 0.1738]	2.21 (36)	0.0332	0.1125	
	Welfare × acute stress	0.0468	[-0.0085, 0.102]	1.72 (36)	0.0945	0.3778	
log(Brain stem 5-HIAA/5-HT)							
	Welfare	-0.0992	[-0.1488, -0.0496]	-4.05 (36)	0.0003	0.0013	**
	Acute stress	-0.0014	[-0.0678, 0.065]	-0.04 (36)	0.9659	1	
	Welfare × acute stress	0.0227	[-0.0215, 0.0668]	1.04 (36)	0.3047	0.6095	
Telencephalon 5-HT							
	Welfare	136.2636	[3.2936, 269.2337]	2.08 (35)	0.0449	0.0898	(*)
	Acute stress	40.3332	[-138.7719, 219.4383]	0.46 (35)	0.6504	1	
	Welfare × acute stress	76.2064	[-42.0107, 194.4234]	1.31 (35)	0.1992	0.5975	
Telencephalon 5-HIAA							
	Welfare	46.7379	[8.8675, 84.6083]	2.51 (35)	0.017	0.0511	(*)
	Acute stress	111.2517	[60.2418, 162.2615]	4.43 (35)	0.0001	0.0005	***
	Welfare × acute stress	59.4963	[25.8276, 93.165]	3.59 (35)	0.001	0.0061	**
Telencephalon 5-HIAA/5-HT							
	Welfare	0.0025	[-0.0039, 0.0088]	0.79 (35)	0.4348	0.4348	
	Acute stress	0.0158	[0.0073, 0.0244]	3.75 (35)	0.0006	0.0032	**
	Welfare × acute stress	0.0057	[1e-04, 0.0114]	2.06 (35)	0.0467	0.2335	

Table S1. Dependent variables (brain stem and telencephalon 5-HT, 5-HIAA, and 5-HIAA/5-HT) and model terms (Welfare: PCA2 dimension 1; Acute stress: PCA2 dimension 2; and Welfare × Acute stress) from post hoc univariate linear models fitted following MANOVA. Linear model statistics are presented as estimates (effect sizes), 95% confidence intervals, t(df) values, raw p-values, Holm-corrected p-values (corrected for multiple testing within each term separately) and significance levels: *** $p < 0.001$, ** $p < 0.01$, * $p < 0.05$, (*) $p < 0.1$.

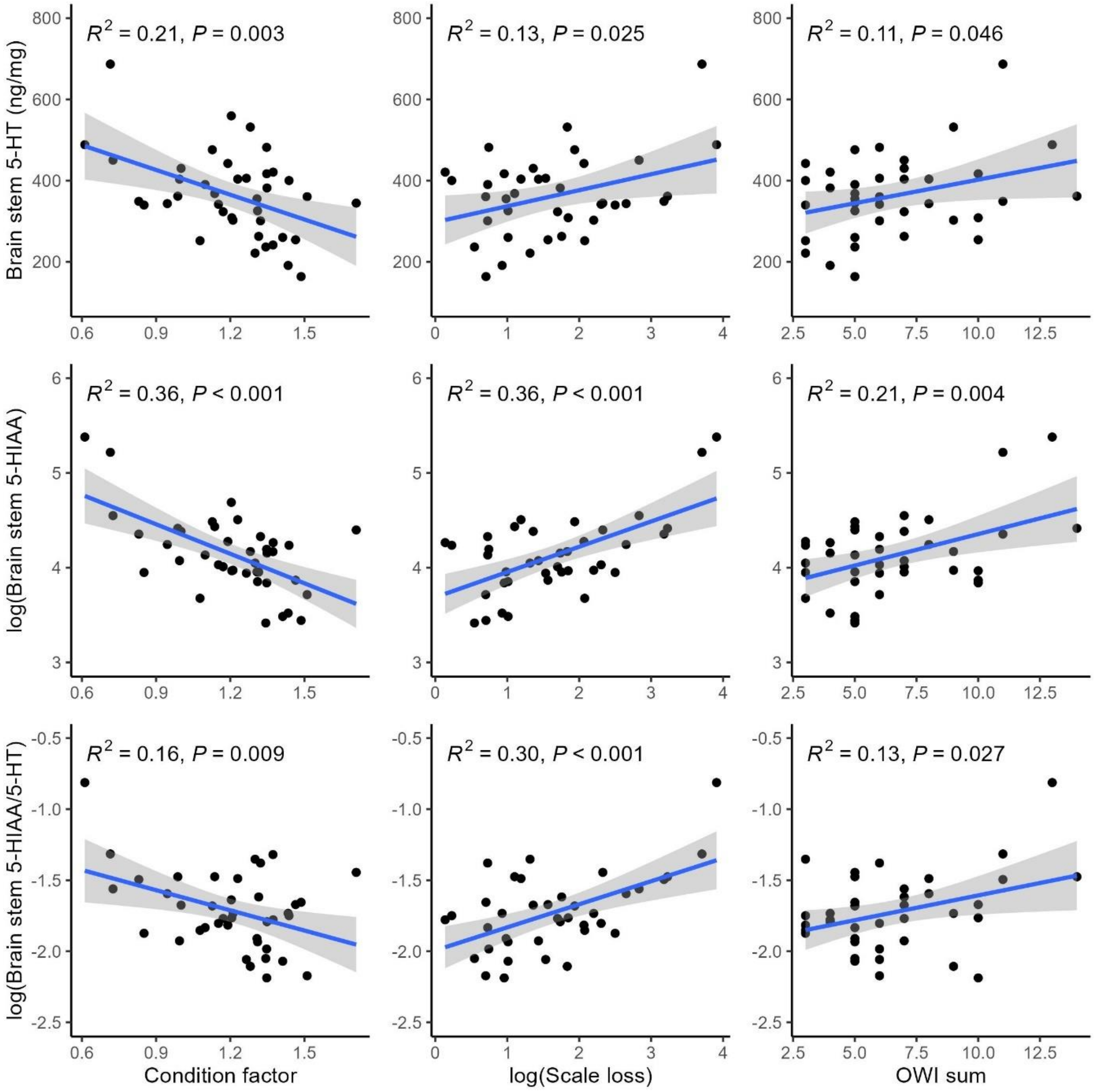

Figure S1. Relationships between welfare markers (condition factor, scale loss, and OWI sum) and brain stem levels of 5-HT, 5-HIAA, and 5-HIAA/5-HT. Scatter plots show least-squares linear regression lines with 95% confidence intervals. Coefficients of determination (R^2) and associated p-values are reported in the panels for descriptive purposes. P-values are not adjusted for multiple testing and should therefore be interpreted with caution. These analyses are provided for data transparency; inference regarding welfare effects is based on PCA, MANOVA, and post hoc linear models (see main text).

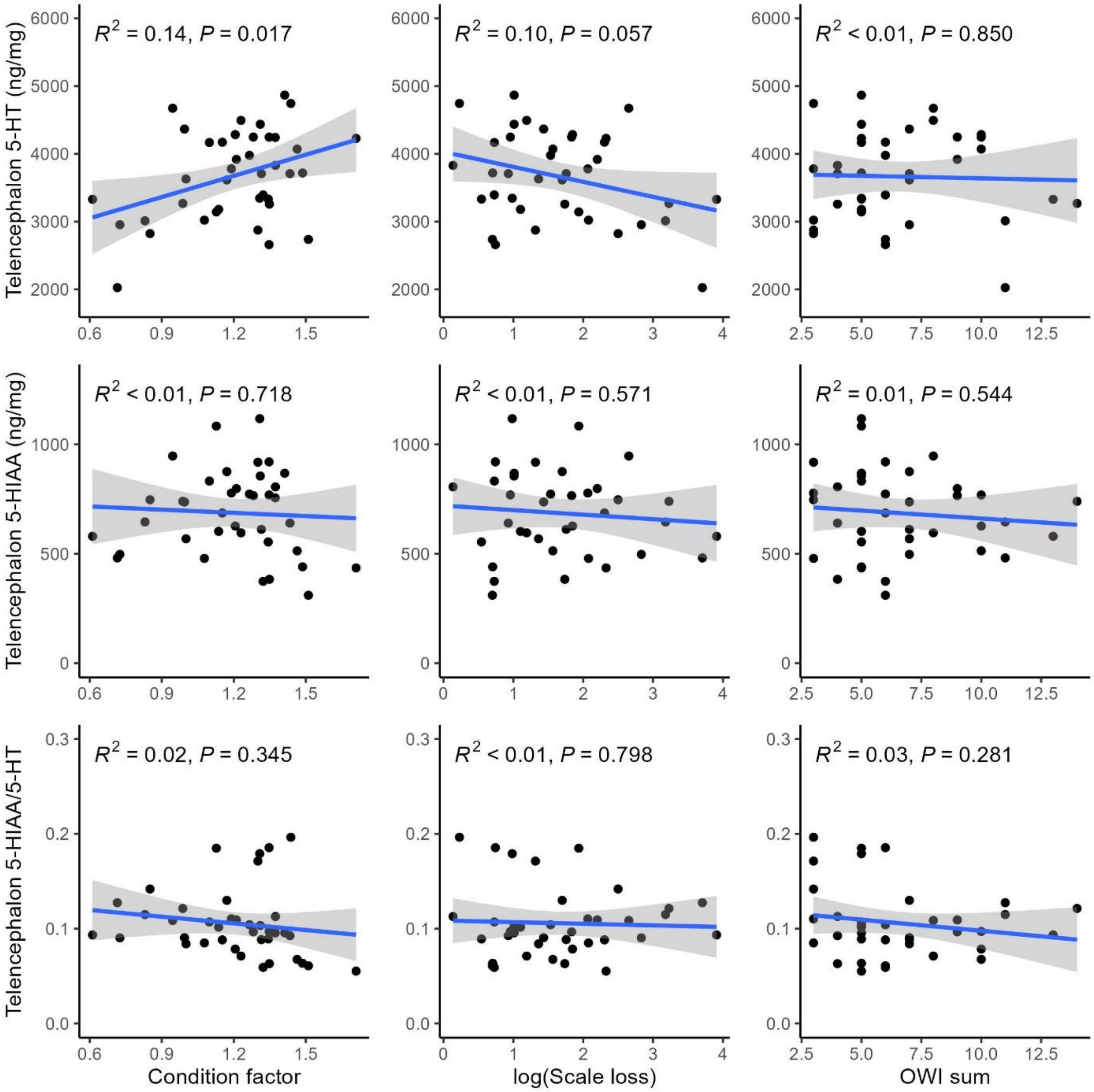

Figure S2. Relationships between welfare markers (condition factor, scale loss, and OWI sum) and telencephalic levels of 5-HT, 5-HIAA, and 5-HIAA/5-HT. Scatter plots show least-squares linear regression lines with 95% confidence intervals. Coefficients of determination (R^2) and associated p-values are reported in the panels for descriptive purposes. P-values are not adjusted for multiple testing and should therefore be interpreted with caution. These analyses are provided for data transparency; inference regarding welfare effects is based on PCA, MANOVA, and post hoc linear models (see main text).

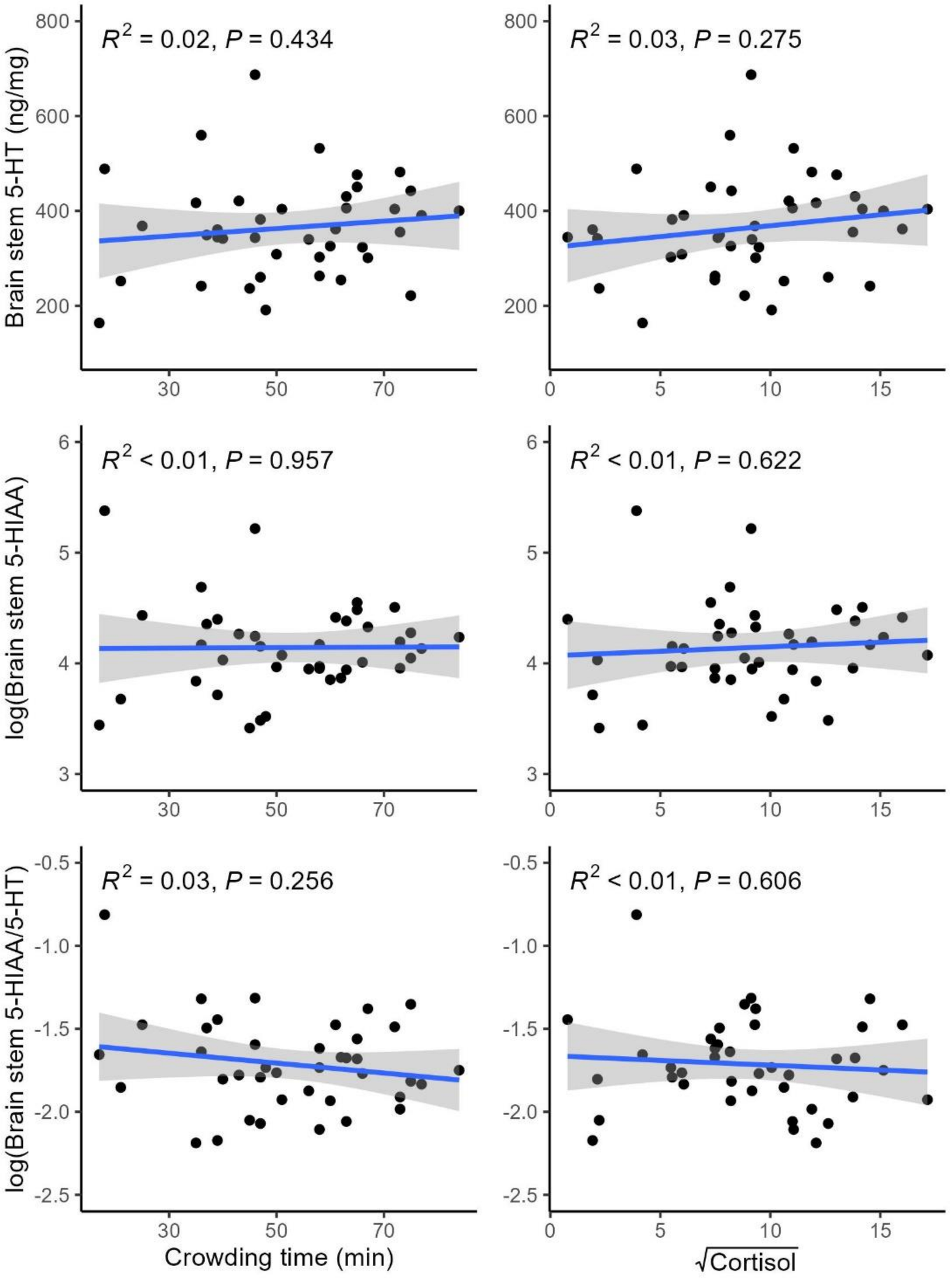

Figure S3. Relationships between acute stress markers (crowding time and plasma cortisol) and brain stem levels of 5-HT, 5-HIAA, and 5-HIAA/5-HT. Scatter plots show least-squares linear regression lines with 95% confidence intervals. Coefficients of determination (R^2) and associated p-values are reported in the panels for descriptive purposes. P-values are not adjusted for multiple testing and should therefore be interpreted with caution. These analyses are provided for data transparency; inference regarding acute stress effects is based on PCA, MANOVA, and post hoc linear models (see main text).

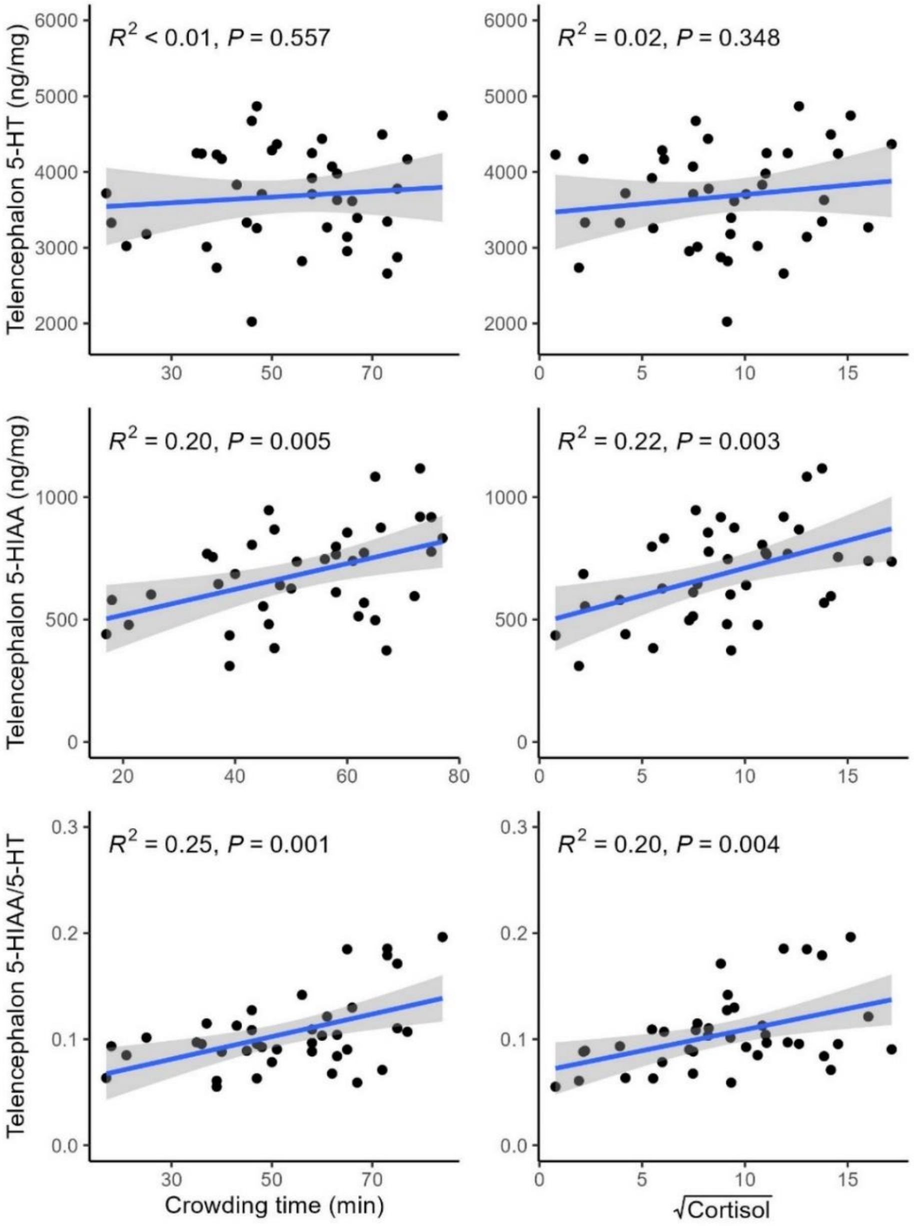

Figure S4. Relationships between acute stress markers (crowding time and plasma cortisol) and telencephalic levels of 5-HT, 5-HIAA, and 5-HIAA/5-HT. Scatter plots show least-squares linear regression lines with 95% confidence intervals. Coefficients of determination (R^2) and associated p-values are reported in the panels for descriptive purposes. P-values are not adjusted for multiple testing and should therefore be interpreted with caution. These analyses are provided for data transparency; inference regarding acute stress effects is based on PCA, MANOVA, and post hoc linear models (see main text).
